# A neuroendocrine role for chemerin in hypothalamic remodelling and photoperiodic control of energy balance

**DOI:** 10.1038/srep26830

**Published:** 2016-05-26

**Authors:** Gisela Helfer, Alexander W. Ross, Lynn M. Thomson, Claus D. Mayer, Patrick N. Stoney, Peter J. McCaffery, Peter J. Morgan

**Affiliations:** 1 Rowett Institute of Nutrition and Health, University of Aberdeen, Foresterhill, Aberdeen AB25 2ZD, Scotland, UK; 2 Faculty of Life Sciences, University of Bradford, Richmond Road, Bradford, BD7 1DP, UK; 3 Biomathematics Statistics Scotland, Rowett Institute of Nutrition and Health, University of Aberdeen, Foresterhill, Aberdeen AB25 2ZD, Scotland, UK; 4 Institute of Medical Sciences, School of Medical Sciences, University of Aberdeen, Foresterhill, Aberdeen AB25 2ZD, Scotland, UK

## Abstract

Long-term and reversible changes in body weight are typical of seasonal animals. Thyroid hormone (TH) and retinoic acid (RA) within the tanycytes and ependymal cells of the hypothalamus have been implicated in the photoperiodic response. We investigated signalling downstream of RA and how this links to the control of body weight and food intake in photoperiodic F344 rats. Chemerin, an inflammatory chemokine, with a known role in energy metabolism, was identified as a target of RA. Gene expression of chemerin (*Rarres2*) and its receptors were localised within the tanycytes and ependymal cells, with higher expression under long (LD) versus short (SD) photoperiod, pointing to a physiological role. The SD to LD transition (increased food intake) was mimicked by 2 weeks of ICV infusion of chemerin into rats. Chemerin also increased expression of the cytoskeletal protein vimentin, implicating hypothalamic remodelling in this response. By contrast, acute ICV bolus injection of chemerin on a 12 h:12 h photoperiod inhibited food intake and decreased body weight with associated changes in hypothalamic neuropeptides involved in growth and feeding after 24 hr. We describe the hypothalamic ventricular zone as a key site of neuroendocrine regulation, where the inflammatory signal, chemerin, links TH and RA signaling to hypothalamic remodeling.

The mechanistic basis of long-term food intake and body weight regulation is poorly understood^1^, yet it underpins key aspects of growth, nutrient partitioning and body composition, including adiposity. Seasonal (photoperiod-sensitive) animals undergo pronounced cycles of weight gain and weight loss together with associated changes in body composition as part of their natural physiology^2^ and it is anticipated that studies of these animals will provide new perspectives on the neuroendocrine basis of long-term body weight cycling and regulation even in humans^3^.

The remarkable cycles in long-term changes in seasonal animals are synchronised by the light:dark cycle (photoperiod). It is now well recognised that the hypothalamic ventricular zone (HVZ) consisting of ependymal cells and tanycytes, is a key site of integration of the photoperiodic signal. Early events in the response involve altered thyroid hormone (TH) and retinoic acid (RA) signalling within the HVZ. The changes are driven by thyroid-stimulating hormone (TSH), derived from the pituitary pars tuberalis, which is released into the median eminence (ME) during long day photoperiod (LD) where it binds to TSH receptors on neighbouring ventricular ependymal cells and tanycytes lining the third ventricle^2^,^4^. TSH controls expression of type 2 deiodinase and, depending on the species, type 3 deiodinase, which together regulate hypothalamic TH availability^5^,^6^,^7^. Hypothalamic RA synthesis and signalling is also under strong photoperiodic regulation within the HVZ^8^ and is directly regulated by TH^9^. It is now generally accepted that changes in hypothalamic TH levels and downstream RA signalling within the neuroendocrine system lead to seasonal changes in body weight, food intake and reproduction^2^,^4^. The big gap in our knowledge has been to understand how changes in hypothalamic TH and RA signalling link to the downstream pathways regulating energy balance in the ARC and other hypothalamic regions.

In terms of neuroendocrine control, the HVZ (ependymal cells and tanycytes) is a relatively unexplored hypothalamic region, yet recent work from divergent fields has highlighted the importance of these cells to neuroendocrine function^10^. For appetite and energy balance, it has become clear that they are a stem cell population that can give rise to new neurons involved in appetite regulation^11^,^12^,^13^. From the seasonal biology perspective, it is also evident that these cells are a key interface between external photoperiodic cues and the long-term changes in metabolic physiology^14^,^15^,^16^,^17^. Additionally, findings from a range of seasonal species point to a potential photoperiodic shift in cellular remodelling involving these cells^10^. In the Syrian hamster (*Mesocricetus auratus*), BrdU incorporation into tanycytes, as a marker for cell proliferation, is affected by photoperiod^18^ and equally in F344 rats and sheep more cells are generated in SD as compared to LD^15^,^17^. In Siberian hamsters (*Phodopus sungorus*) and sheep, adaptations of neural pathways as well as cell structure and morphological changes in the hypothalamus are also associated with changes in photoperiod^14^,^16^,^19^,^20^. Taken together these data suggest that structural changes within the hypothalamus may be at the core of the long-term effects of photoperiod on physiology, such as regulation of energy balance and body weight.

In this study, we investigated signalling downstream of RA in the HVZ and identified *Rarres2*, encoding the inflammatory chemokine chemerin, as a key gene expressed in tanycytes and ependymal cells of the HVZ, which is responsive to both photoperiod and RA. Chemerin (*Rarres2*), a recently discovered adipokine with numerous biological functions^21^, is the ligand for the G-protein coupled receptors Cmklr1, Gpr1 and Ccrl2^22^. It has been suggested that chemerin has a biological role in obesity, because in humans serum chemerin levels correlate with body mass index and other obesity-related biomarkers^23^,^24^,^25^,^26^,^27^. In mice, serum chemerin levels are upregulated by high-fat diet feeding^28^ and *Cmklr1* knockout mice have reduced body weight and food intake compared to wild-type mice independently of diet^29^,^30^. However, a central role of chemerin has not been demonstrated. In this study we provide the first evidence that chemerin in the HVZ plays a pivotal role in energy balance regulation.

## Results

### Chemerin is a downstream effector of retinoic acid in the hypothalamus

**Figure 1:**
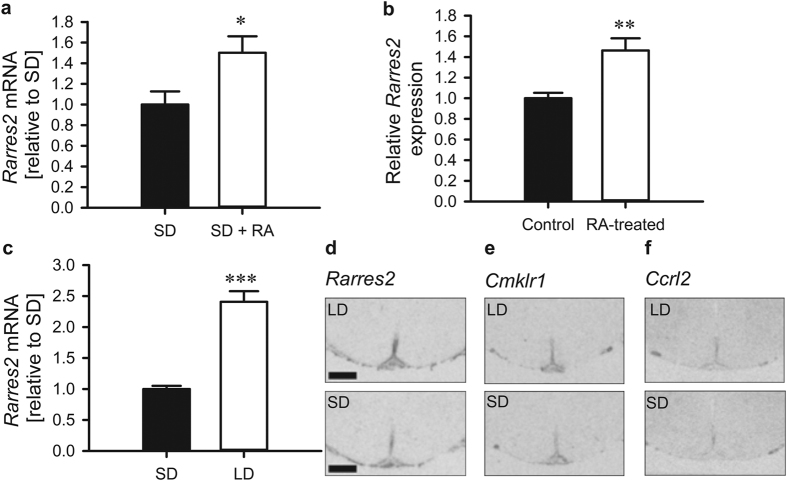
Chemerin (*Rarres2*) is a photoperiod-sensitive signal downstream of retinoic acid in the hypothalamus. (**a**) *Rarres2* mRNA expression in male F344 rats 24 hrs after ICV RA injection (10 μM, n = 5) is significantly increased compared to vehicle treated rats (n = 6). F344 rats were acclimated in a SD (8 h:16 h) light:dark schedule before RA injection. (**b**) *Rarres2* mRNA expression is also responsive to RA *ex-vivo*. Hypothalamic slices from Sprague Dawley rat pups were cultured for 4 days and then treated for 48 hrs with 1 μM RA (n = 11/group) before qPCR analysis. (**c**) *Rarres2* mRNA is significantly increased by 28 days of exposure to LD (16 h:8 h; n = 8) compared to SD (n = 8). (**d**) *Rarres2* expression is localised in the ependymal cell layer and median eminence of the hypothalamus, the same neuroanatomical regions as its receptors (**e**) *Cmklr1* and (**f**) *Ccrl2*. Representative autoradiographic images are shown. Scale bar is 1.0 mm. Data presented are mean ± SEM, *p < 0.05, **p < 0.01, ***p < 0.001. LD, long day; SD, short day; RA, retinoic acid.

To identify genes that mediate the downstream effect of RA in the hypothalamus, we performed an Agilent microarray analysis covering 30003 genes on hypothalamic blocks of F344 rats administered ICV with exogenous RA. We found the most significant changes in genes related to inflammatory pathways (Table 1) and of these genes, a promising candidate was *Rarres2*, which encodes chemerin (1.41-fold increase, p < 0.001). Using *in-situ* hybridisation, we confirmed the microarray results and showed that *Rarres2* mRNA levels were significantly increased in response to RA injection in the HVZ (1.50-fold, *p* = 0.034; Fig. 1a). *Ex-vivo*, real-time PCR revealed that RA also induced significant upregulation of *Rarres2* mRNA in hypothalamic slice cultures of Sprague-Dawley rats (1.46-fold, *p* = 0.006; Fig. 1b). To determine chemerin’s candidacy as a mediator of photoperiodic responses in F344 rats, we next asked whether its expression was changed by photoperiod in the relevant regions of the hypothalamus. *Rarres2* mRNA expression was significantly increased in the HVZ by 28 days exposure to LD as compared to SD (2.41-fold, *p* < 0.001; Fig. 1c) and found in the ependymal cell layer lining the third ventricle and the ME of the hypothalamus (Fig. 1d). Chemerin is known to bind to three G-protein coupled receptors^26^, Cmklr1 (ChemR23, DEZ), Ccrl2 and Gpr1. Like *Rarres2*, *Cmklr1* and *Ccrl2* mRNAs are also localised in the ependymal cell layer and ME of the hypothalamus (Fig. 1e,f), but we were unable to detect expression of *Gpr1* mRNA in the hypothalamus of F344 rats by *in-situ* hybridisation (data not shown).

### Long-term chemerin infusion modulates food intake

To explore whether chemerin has an impact on physiological function, we first tested the effect on body weight and food intake of chronic delivery of chemerin over 28 days into F344 rats held in SD. Given that RA levels are elevated in LD, our hypothesis was that chemerin would induce elevated levels of body weight and food intake in the long-term.

**Figure 2:**
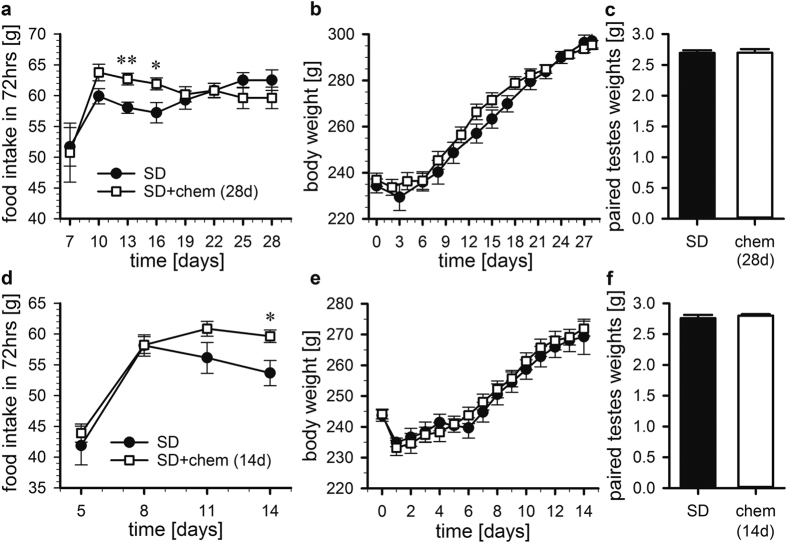
Effect of chronic ICV infusion of chemerin on food intake, body weight and testicular growth. Ad libitum fed male F344 rats, held in a SD (8 h:16 h) light:dark schedule, received a chronic ICV infusion of chemerin (100 nmol/day; n = 7) or vehicle (n = 7) for 28 days (top panels; **a–c**) or 14 days (lower panels; **d–f**). Food intakes (**a,d**), body weights (**b,e**) and paired testes weights are shown (**c,f**). Data shown are mean±SEM; *p < 0.05, **p < 0.01. SD, short day; chem, chemerin.

Initially, a range of doses (100 nmol, 1 μmol, 10 μmol) of human chemerin dissolved in 5 μl saline injected ICV into F344 rats was tested and the strongest response was found at a concentration of 100 nmol (data not shown). Therefore, all subsequent experiments were performed at this concentration. Following chronic chemerin infusion (100 nmol/day for 28 days), rats exhibited a period of higher food intake. 2-way ANOVA showed that there was a significant interaction between treatment and day (*p* = 0.001) and pairwise comparison revealed that rats infused with chemerin ate significantly more on day 12 and 15 than their saline-infused counterparts (*p* = 0.004 and *p* = 0.030, respectively). However, the effect of chemerin on food intake was temporary and at 28 days no significant difference was observable between the two groups (Fig. 2a). The transient increase of chemerin on food intake appeared to be associated with a transient effect on body weight, however, this narrowly failed to reach statistical significance (2-way ANOVA, *p* = 0.059; Fig. 2b).

In a repeat study, chemerin was infused for 14 days (100 nmol/day). Food intake resembled that of the original 28 day experiment with significantly higher food intake in chemerin-infused rats on day 14 (*p* = 0.015; Fig. 2d), but no differences in body weight was observed in this shorter study (*p* = 0.636; Fig. 2e). The repeat was performed to facilitate analysis of the effect of chemerin in the brain at a time point when the physiological changes were manifested, based on the results from the first experiment.

Chemerin treatment had no effect on paired testes weight in either experiment (28 days: *p* = 0.957, Fig. 2c; 14 days: *p* = 0.489, Fig. 2f).

### Acute impact of chemerin on physiological function

**Figure 3:**
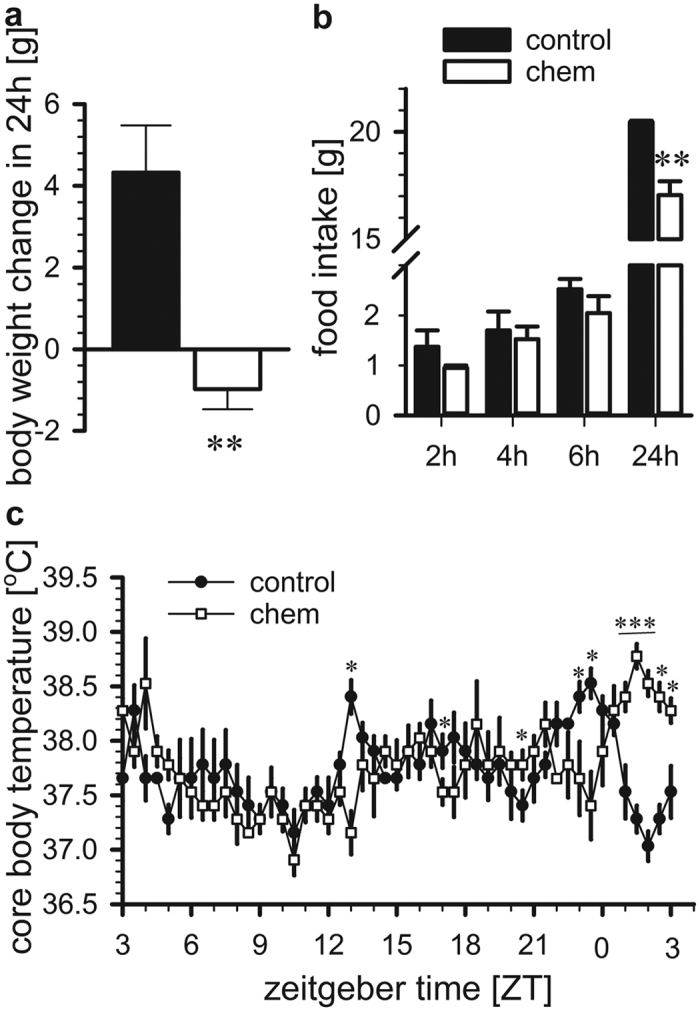
Effect of acute ICV injection of chemerin on body weight, food intake and body temperature. Ad libitum fed F344 rats held in a 12 h:12 h light: dark schedule received an ICV injection of 100 nmol chemerin (n = 4) or vehicle (n = 4) at ZT3. 24 hrs after the injection (**a**) body weight and (**b**) food intake was significantly decreased compared to saline-injected rats. (**c**) Core body temperature was significantly different in chemerin-injected rats compared to saline-injected rats. Data shown are mean ± SEM, *p < 0.05, **p < 0.01, ***p < 0.01. Chem, chemerin.

As a next step, we investigated the acute effects of chemerin on body weight and food intake. 100 nmol of chemerin was administered ICV at ZT3 into F344 rats as a bolus injection. Acute injection of chemerin had an opposite effect on physiology compared to long-term infusion. Pairwise comparison revealed that acute chemerin injection caused a marked reduction in body weight (*p* = 0.006; Fig. 3a). The change in body weight was accompanied by a significant decrease in food intake after 24 hrs (*p* = 0.002). No difference was detected in food intake over a shorter time period (2, 4 and 6 hrs; Fig. 3b). Core body temperature was significantly different in chemerin-injected rats compared to saline-injected rats (two-way RM ANOVA, treatment x time interaction, p < 0.001). Pairwise comparison showed that the maximum difference in body temperature, with an increase of about 1.5 °C, was by 23 hrs post-injection (p < 0.001; Fig. 3c). However, the mean body temperature over the entire 24 hr period was not statistically different between treatments (t-test, *p* = 0.828).

### Chemerin alters hypothalamic neuropeptide expression in the short-term

**Figure 4:**
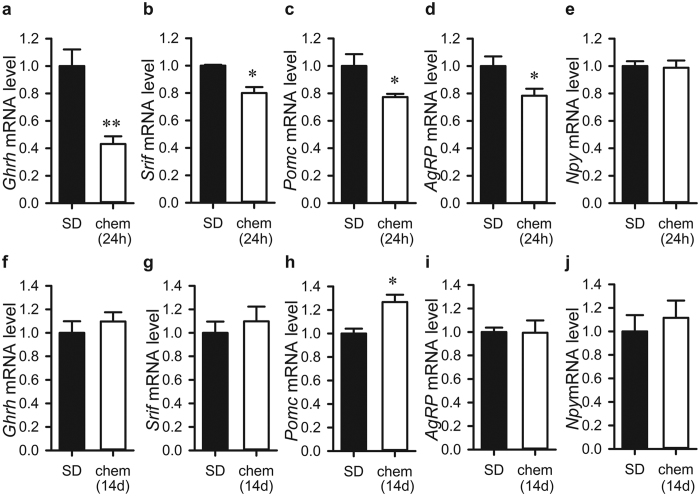
Effect of chemerin administration on gene expression of growth and feeding-related hypothalamic peptides. (**a**) *Ghrh*, (**b**) *Srif*, (**c**) *Pomc* and (**d**) *AgRP* mRNA levels were significantly downregulated 24 hrs after a bolus ICV injection of chemerin (100 nmol; n = 4) compared to vehicle injection (n = 4) whereas (**e**) *Npy* mRNA expression was unaffected. (**f–j**) Chronic infusion of chemerin (100 nmol/day/14 days, n = 7) had no effect on (**f**) *Ghrh*, (**g**) *Srif*, (**i**) *AgRP* and (**j**) *Npy* mRNA levels compared to vehicle infusion (n = 7), but (**h**) *Pomc* mRNA expression was significantly upregulated. Data shown are mean ± SEM and presented relative to SD; *p < 0.05, **p < 0.01. Chem, chemerin.

Next, we tested if chemerin was involved in the regulation of hypothalamic peptidergic modulators that play a role in growth and feeding (Fig. 4). Hypothalamic neuropeptide gene expression was investigated in rats acutely injected with chemerin for 24 hrs (Fig. 4a–e) and in rats chronically infused with chemerin for 2 weeks (Fig. 4f–j). In line with chemerin’s potential to suppress body weight in the short-term, *Ghrh* and *Srif* mRNA expression were significantly lower in chemerin-injected rats than in SD-housed control rats (*p* = 0.005 and *p* = 0.012, Fig. 4a,b, respectively). *Pomc* and *AgRP* mRNA levels were also significantly down-regulated by acute chemerin injection (*p* = 0.05 and *p* = 0.032, Fig. 4c,d, respectively), while no difference was found in *Npy* mRNA expression (*p* = 0.931, Fig. 4e). Chronic delivery of chemerin for 2 weeks had no effect on *Ghrh* (*p* = 0.457, Fig. 4f), *Srif* (*p* = 0.544; Fig. 4g), *AgRP* (*p* = 0.955; Fig. 4i) and *Npy* (*p* = 0.580; Fig. 4j), but *Pomc* mRNA expression was significantly upregulated compared to saline-infused rats (*p* = 0.0105, Fig. 4h).

### Chemerin drives changes in hypothalamic plasticity

**Figure 5:**
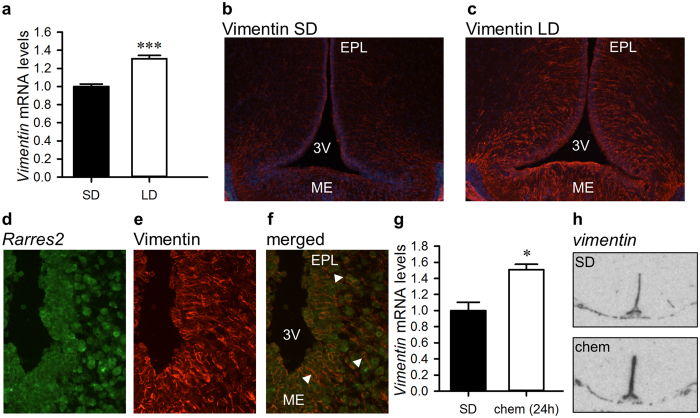
Chemerin drives hypothalamic remodelling. (**a**) *Vimentin* mRNA in the ependymal cell layer is significantly increased by 28 days of exposure to LD (n = 8) compared to SD (n = 8). (**b**) Vimentin immunostaining is reduced in the dorsal part of the ependymal cell layer in F344 rats held in SD (8 h:16 h) compared to (**c**) F344 rats held in LD (16 h:8 h) photoperiod for 28 days (magnification 100x; Vimentin in red overlaid with Dapi in blue). (**d**) *Rarres2* mRNA (green) and (**e**) Vimentin immunolabelling (red) are (**f**) coexpressed in the ME and the EPL (examples indicated by white arrowheads; magnification 400x). (**g**) *Vimentin* mRNA is upregulated in F344 rats 24 hrs after a bolus ICV injection of chemerin (100 nmol; n = 4) relative to controls (n = 4). (**h**) *Vimentin* mRNA expression in the tanycytes and ependymal cells lining the third ventricle of the hypothalamus in vehicle- and chemerin-injected F344 rats. Representative images are shown. Data presented are mean ± SEM, *p < 0.05, ***p < 0.001; 3 V, third ventricle; chem, chemerin; EPL, ependymal cell layer; ME, median eminence; LD, long day; SD, short day.

The next experiment was to investigate whether ependymal cell and tanycyte morphology was altered by chemerin. We used the intermediate filament protein vimentin, which shows a marked photoperiodic difference in hamsters^14^,^20^,^31^, as a marker for estimating hypothalamic plasticity, which was defined as the capacity of the brain to adjust its cell structure and morphology^16^. First we confirmed that vimentin mRNA and protein expression also changes photoperiodically in the HVZ of F344 rats. *Vimentin* mRNA was significantly up-regulated in the ependymal cell layer in LD-housed rats compared to their SD-housed counterparts (1.31-fold, p > 0.001; Fig. 5a). Vimentin-positive cells were found in the ependymal cells and the tanycytes lining the third ventricle as well as in the ME. The most pronounced photoperiodic difference was found in the ependymal cell layer. While in LD, vimentin-positive cells where densely distributed forming long processes reaching into the medial basal hypothalamus, these were decreased or absent in SD in the ependymal cell layer (Fig. 5b,c). Using a fluorescence *in-situ* hybridisation (fISH) for *Rarres2* followed by immunostaining for vimentin we confirmed that *Rarres2* mRNA is expressed by tanycytes. *Rarres2* mRNA colocalised with vimentin in cells in the HVZ and ME (Fig. 5d–f).

Next, we investigated if vimentin expression changes after chemerin injection. By 24 hrs post chemerin injection, *vimentin* mRNA was increased by about 50% in the ependymal cell region of chemerin-injected F344 rats relative to controls (*p* = 0.0257, Fig. 5g,h), indicating that one of chemerin’s initial actions in the hypothalamus might be as a driver of structural remodelling of the hypothalamus.

**Figure 6:**
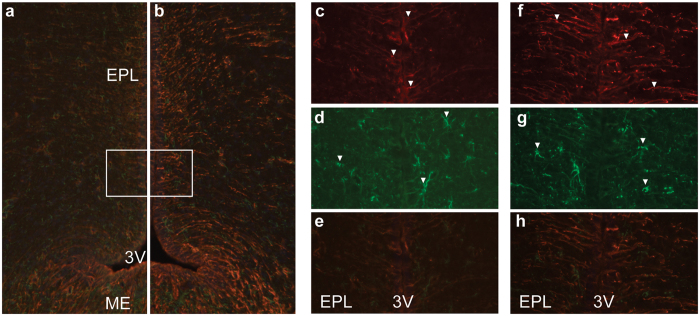
Vimentin and glial fibrillary acidic protein (GFAP) immunostaining in the rat hypothalamus. F344 rats held in a SD (8 h:16 h) light:dark schedule were infused with (**a**) vehicle (n = 5) or (**b**) 100 nmol/day/14 days chemerin (n = 5). (**a,b**) Merged overview of the relevant hypothalamic region with vimentin in red, GFAP in green and nuclear counterstaining with DAPI in blue (magnification 100X). (**c–h**) Representative images of inset outlined in white in (**a,b**) showing the EPL lining the 3 V of the hypothalamus (magnification 400X). Vimentin immunolabelling (red) is lower in the dorsal part of the ependymal region of vehicle (**c**) compared to chemerin infused rats (**f**). GFAP-positive cells (green) are found throughout the hypothalamus with no difference between (**d**) vehicle and (**g**) chemerin infused rats. (**e,h**) Colocalisation of vimentin (red) and GFAP (green) shows that there is no overlap. EPL: ependymal cell layer; 3 V: third ventricle; ME: median eminence. Examples of positive cells are indicated by white arrowheads.

Because the effect on the mRNA level was not maintained over the long-term (data not shown), we used immunohistochemistry to label ependymal cells to investigate if morphological changes were evident. In the control rats, little to no vimentin immunoreactivity was detected in the ependymal cells and tanycytes, with clearly fewer stained cells compared to chemerin-infused rats (Fig. 6a,c), similar to SD photoperiod. The difference in staining was most prominent in the dorsal part of the ventricle. In chemerin-infused rats, vimentin immunostaining revealed intensely stained cells in the ependymal cells and in the tanycytes in ventral and dorsal regions of the third ventricle. Vimentin-positive cells extended away from the ependymal cell layer appearing densely distributed with longer processes (Fig. 6b,f), resembling LD conditions. Vimentin immunostaining was also strongly observed in the ME, but equally so in chemerin-infused and control animals (Fig. 6a,b). Glial fibrillary acidic protein (GFAP)-positive cells, indicative of astrocytes, were found throughout the hypothalamus with no apparent difference between vehicle and chemerin-infused F344 rats (Fig. 6d,g). Colocalisation of vimentin and GFAP showed no overlap (Fig. 6e,h).

## Discussion

**Figure 7:**
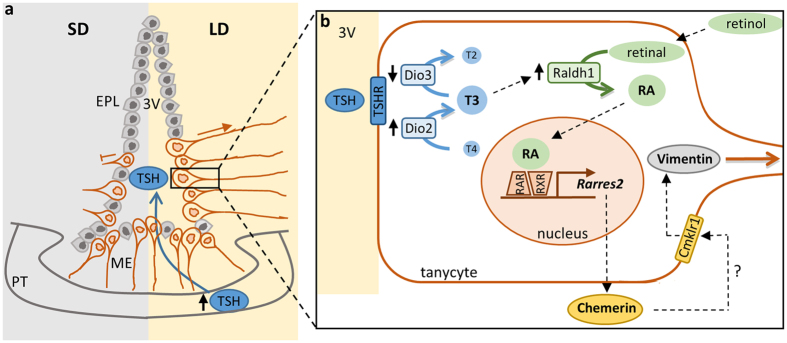
Hypothetical model of how chemerin might contribute to morphological changes in the hypothalamus. (**a**) During long day (LD) photoperiod, thyroid-stimulating hormone (TSH) is released from the pars tuberalis (PT) into the median eminence (ME) where it binds to TSH receptors (TSHR) on tanycytes lining the third ventricle (3 V). In LD, densely distributed tanycytes form long projections into the medial basal hypothalamus, which decrease or disappear in short day (SD) photoperiod. (**b**) TSH binds to TSHR on tanycytes and this increases Dio2 expression and decreases Dio3 expression. Dio2 converts thyroid hormone T4 into the more active form T3, which is then inactivated by Dio3. In LD, rising levels of T3 upregulate Raldh1 expression which synthesises retinoic acid (RA). RA enters the nucleus where binding to retinoic acid receptors (RAR/RXR) leads to transcription of Rarres2 and other target genes. Chemerin (Rarres2) binds to its receptors (Cmklr1) possibly on the same or on neighbouring cells. An increase in chemerin results in an increase in Vimentin in the cell and the tanycyte extends away from the ependymal cell layer (EPL) into the hypothalamus forming long projections during this process.

Previous studies have shown the importance of local synthesis of both TH and RA within the HVZ of the hypothalamus to the photoperiodic response^2^,^4^ and recent work has revealed that TH regulates the expression of Raldh1 suggesting that TH drives the photoperiodic response at least in part through RA signalling^9^ (Fig. 7). In this study, the expression of the inflammatory chemokine chemerin and its receptors, Cmklr1 and Ccrl2, were localised to the HVZ and it was shown that chemerin (*Rarres2*) gene expression was responsive to photoperiod and RA. Given that chemerin infused ICV into SD rats can partially and transiently mimic the SD to LD transition in terms of food intake, these findings show that chemerin, and intermediate filament protein, vimentin, are downstream elements of the hypothalamic RA pathway. Moreover, these data provide the first plausible evidence to link photoperiod to the long-term regulation of energy balance and body weight through potential remodelling of the hypothalamus. The basis for this conclusion is that previous studies in Siberian hamster have shown how cells immunopositive for vimentin project radially from tanycytes into the medial basal hypothalamus, although the extent of the radial projections are photoperiod-dependent, with long projections under LD, which decrease or disappear under SD^20^. Other work undertaken in hamsters and sheep has also shown morphological changes in the hypothalamus that are associated with changes in photoperiod^14^,^16^,^18^,^19^. Thus, the increased vimentin immunolabelling of projections from the tanycytes and ependymal cells of the HVZ of SD-housed F344 rats in response to 2 week infusion of chemerin mimics a LD response. From this it seems reasonable to conclude that the changes in vimentin immunoreactive projections from the tanycyte and ependymal cells contribute to the mechanism involved in the neuroendocrine changes in physiology, such as food intake and body weight. It also provides further evidence to support the emerging importance of the HVZ as a key site of regulation within the neuroendocrine hypothalamus (Fig. 7).

Chemerin is an adipokine encoded by the gene *Rarres2*, originally identified by its RA responsiveness in psoriatic skin lesions^21^. It has since been found to have a number of potential roles including chemotactic, adipokine, autocrine/paracrine, adipogenic, angiogenic and reproductive functions^22^,^26^. Nonetheless, its role in the regulation of energy metabolism and metabolic syndrome^23^,^26^,^28^,^29^,^32^ makes it a promising candidate relevant to our studies of long-term energy balance regulation.

In this study we show that chemerin induces a transient increase in food intake, but this effect is not prolonged. The most likely explanation for this transient effect is that constant infusion of chemerin ICV may only poorly mimic the pattern of secretion and bioavailability of endogenous chemerin over longer timeframes. For example, it is known that chemerin must be enzymatically cleaved to become bioactive and a number of isoforms of chemerin with varying receptor affinity have been detected in different tissues^26^. Secondly, prolonged exposure to high levels of chemerin might cause desensitisation of its receptors and/or cause habituation. Nevertheless, while chemerin partially mimics the photoperiodic SD to LD transition when infused into the third ventricle of animals in SD, a very different response was observed when given as an acute bolus injection ICV into rats on a 12 h:12 h light:dark schedule, a photoperiod that maintains rats in a LD state^33^. Here, both food intake and body weight, were suppressed within 24 hrs. This indicates an anorectic effect, which differs to a previous report where chemerin was injected directly into the hypothalamus and yet no effect on food intake and body weight was observed^34^. The difference in response may be explained by the different sites of delivery (ARC tissue vs ICV) and doses used.

The apparent bimodal response to chemerin seen in this study, when given either acutely or chronically, is also most likely related to the route, mode of delivery and/or dose used, which would impact on chemerin’s bioavailablity or receptor sensitivity as suggested above. However another factor could be photoperiod. The chronic infusion study was designed to replicate the SD to LD transition. Based on levels of chemerin gene expression in the hypothalamus, it was predicted that hypothalamic chemerin levels would be considerably lower under SD than LD. On this basis infusion of chemerin into rats in SD would be against a low background level of chemerin in the hypothalamus. Infusion of chemerin into LD animals was not tested, as it was anticipated that this would be without effect. However, it is possible that the effect of acute administration of chemerin to rats on 12 h:12 h photoperiod reflects a true LD response, where chemerin becomes inhibitory when given at high dose to rats with already high endogenous levels of chemerin. In that regard it is noteworthy that, in another study, high doses of chemerin injected intra-peritoneally for 17 days into Wistar rat on 12 h:12 h photoperiod suppressed both food intake and body weight^35^.

Chemerin exerts its effects by binding to and activating the G protein-coupled receptor Cmklr1 but it also serves as a ligand for the chemokine receptor-like protein Ccrl2 and the G protein-coupled receptor Gpr1^26^. Both, *Cmklr1* and *Ccrl2* mRNAs were expressed in the ependymal cell layer of the HVZ, closely matching the distribution of *Rarres2* mRNA. Ccrl2 is a non-signalling receptor that binds chemerin to increase its concentration^28^ therefore, since chemerin is in part locally produced, it might function to amplify the hypothalamic activity of chemerin. Little is known about Gpr1, although it has recently been associated with high-fat diet induced glucose intolerance^36^. By *in-situ* hybridisation, we could not detect *Gpr1* mRNA expression in the relevant hypothalamic regions associated with energy balance control. Since loss of Gpr1 does not affect body weight in *Gpr1* knockout mice on a chow diet^36^, the neuroendocrine effects of chemerin are likely to be predominantly regulated via the chemerin/Cmklr1 axis.

Chemerin’s role in the photoperiod response, and thereby its potential link to energy balance, was discovered following acute injection of RA into the third ventricle of F344 rats in SD, which increased the expression of chemerin, consistent with higher production of chemerin under LD. The acute injection of RA into the third ventricle of F344 rats on SD revealed changes not only in chemerin, but also altered expression, within the hypothalamus, of a number of other genes associated with inflammatory and immune responses (Table 1). Several studies have implicated inflammation in key hypothalamic areas in the control of body weight, with the inflammation involved being largely a pathophysiological consequence of overnutrition^37^,^38^. In addition, it has been suggested that this may be a cause of the increase in the biologically defended level of body weight that underlies obesity^38^. The finding that chemerin can alter long-term food intake in the seasonal F344 rats provides an example of a physiological, rather than pathophysiological, role for an inflammatory signal in the neuroendocrine control of appetite and energy balance. In this context, it will be interesting to study if the other genes, uncovered by the microarray analysis, are also involved in the photoperiod response and whether their effect is a physiological rather than a pathophysiological response.

It is well established that feeding behaviour is under the control of hypothalamic circuits^39^. Despite this, chemerin does not seem to act directly through known neuroendocrine energy balance and growth pathways to exert its effects. Following acute bolus injection of chemerin into rats in 12 h:12 h photoperiod, food intake and body weight are reduced. Yet gene expression in the ARC for both orexigenic (*AgRP)* and anorexigenic (*Pomc),* as well as stimulators *(Ghrh*) and inhibitors *(Srif)* of growth, are all reduced. This is against expectation, as these opposing signals are typically reciprocally regulated^39^. Similarly, in response to long-term chemerin-infusion into SD rats, the pattern of gene expression of hypothalamic neuropeptides did not follow simple prediction according the status of energy balance. Thus consistent with previous studies of energy balance in seasonal animal models, expression of hypothalamic orexigenic and anorexigenic genes is not a straightforward reflection of food intake or body weight status of the animal^40^,^41^. However, this is not something restricted to seasonal animal models, as in mice with a genetic ablation of Cmklr1, hypothalamic *AgRP*, *Pomc* or *Npy* expression also do not correlate with the levels of food intake^29^. Therefore, it seems that the long-term effect of chemerin on food intake and body weight is not a direct or simple correlative neuroendocrine relationship with known appetite and energy balance pathways and so other hypothalamic mechanisms must be involved in the effects of chemerin on food intake and body weight. A more tantalizing possibility is for chemerin to exert its effects through cellular remodelling. The effect of chemerin on *vimentin* mRNA levels within 24 hrs and vimentin immunolabelling over 2 weeks within the cells of the HVZ lends credence to such an idea. From the seasonal biology perspective, this study provides further evidence for the view that photoperiod dependent changes in tanycytes and ependymal cells drive long-term changes in metabolic physiology^10^,^42^.

In conclusion, we have identified a novel signalling cascade, downstream of TH and RA, in the hypothalamus of seasonal rats. Specifically we show that the inflammatory gene, Rarres2, encoding the chemokine chemerin, has restricted expression in the HVZ (both tanycytes and ependymal cells) and it has a role in the regulation of food intake and body weight in seasonal animals. This involves chemerin-induced changes in vimentin expression showing that the seasonal neuroendocrine response involves hypothalamic remodelling. Given that the components of the signalling pathway described in this study are also expressed in the HVZ of non-seasonal animals, it is likely that these findings will have relevance to the regulation of food intake and energy balance in mammals more widely.

## Methods

### Animals

Animal experiments were approved by the ethics committee at the Rowett Institute of Nutrition and Health and carried out in accordance with UK Home Office guidelines, licensed under the Animals (Scientific Procedures) Act, 1986 (project licence numbers PPL60/3615 and PPL60/4282).

*In-vivo* experiments used male F344/NHsd rats from Harlan Sprague-Dawley Inc. Rats were acclimatised for 10–14 days before they were randomly assigned to weight-matched experimental groups. *Ex-vivo* experiments used male Sprague Dawley rat pubs bred in the animal facility at the University of Aberdeen.

Besides from photoperiod (SD 8 h:16 h; LD 16 h:12 h) changes, rats were kept under the same standard conditions (ambient temperature: 21 ± 2 °C; relative humidity: 55 ± 10%; average light intensity: 150 lux). Rats were housed in standard rat cages type RC2/f with G6 woodchip bedding and shredded paper and a red plastic tunnel for enrichment. Rats had unrestricted access to food (CRM(P) Rat and Mouse Breeder and Grower, standard pelleted diet, Special Diet Services) and water. For central administration of substances, cannulae were stereotaxically implanted into the ventricle as described previously^7^.

Before killing, rats were assigned random numbers to ensure blind analysis for the experimental conditions. For *in-situ* hybridisation studies, rats were killed with isoflurane inhalation followed by decapitation and for immunohistochemistry studies, rats were killed with intraperitoneal injections of Pentoject (Animalcare Limited) followed by transcardially perfusion with 4% paraformaldehyde in 01.M phosphate buffer (PB). For all experiments killing took place at around zeitgeber time (ZT) 3.

### Retinoic acid (RA) injections

F344 rats were group housed and exposed to SD during acclimatisation. Rats (weight range 201.7–229.6 g) were surgically prepared and intracerebroventricular injections (ICV) were performed as described previously^7^. Stereotaxic coordinates to reach the third ventricle of the hypothalamus were 0.8 mm posterior to the bregma line on the mid-sagittal line, 6.5 mm below the outer surface of the scull. After surgery, rats were pair-housed. Cannula placement was confirmed by a positive dipsogenic response to 100 ng of angiotensin (Sigma-Aldrich) in 5 μl of 0.9% saline. 7 days later, rats that tested positive to angiotensin, were injected with 5 μl of 10^−4^ M all-*trans* RA in 10% DMSO in 0.9% saline (n = 4) or vehicle (n = 4) at ZT3. 24 hrs after RA injections, rats were killed. This experiment was repeated to provide tissue for *in-situ* hybridisation analysis (repeat study weight range 185.3–212.2 g). Here, rats were injected with 5 μl of 10^−5^ M all-*trans* RA in 10% DMSO in 0.9% saline (n = 5) or vehicle (n = 6).

### Chemerin injections

F344 rats were exposed to a 12 h:12 h schedule and pair-housed until ICV injections. The surgery weight of the rats was 190.4–211.7 g. Initially, a chemerin dose-response experiment was performed. Rats were injected with 100 nmol (n = 4), 1μmol (n = 5) or 10 μmol (n = 4) human chemerin (Cell guidance systems) in 0.9% saline or vehicle (n = 4) at ZT3. After chemerin injections rats were single housed and presented with a preweighed amount of chow and food intake was measured after 2, 4, 6 and 24 hrs. Body weight was measured before ICV injection and 24 hrs afterwards. Core body temperature was measured remotely using i-button^®^ devices (Maxim Integrated Products) implanted into the peritoneum during ICV cannulation. Rats were killed 24 hrs after chemerin injection at ZT3.

### Chemerin infusion

F344 rats were group housed in SD from arrival. After acclimatisation, brain infusion cannulae were implanted into the third ventricle of the rats (weight range of 227.5–253.2 g). Based on the results of the dose-response experiment, 100 nmol/day of human chemerin in 0.9% saline (n = 7) or vehicle (n = 7) was constantly delivered via subcutaneously implanted osmotic minipumps (Alzet; model 1004, flow rate 0.11 μl/hr) connected to the ICV cannula over a 28 day period. For accurate body weight and food intake measurements (at least 3× weekly), rats were housed singly after surgery. Cannulae placement was verified at the end of the experiment by dye administration. After 28 days, rats were killed, brains collected and paired testes weight recorded.

The study was repeated with an infusion period of 14 days. The weight range of the repeat study was 232.6–254.9 g. Alzet osmotic minipumps model 1002, flow rate 0.25 μl/hr were used. 14 days after surgery, rats were killed and brains collected for *in-situ* hybridisation (n = 7/group) and immunohistochemistry (n = 5/group).

### Photoperiod study

After acclimatisation in 12 h:12 h, F344 rats (weight range of 117.1–142.7 g) were randomly divided into two weight matched groups (n = 8/group) and transferred to either SD or LD. After 28 days, single-housed rats were killed at ZT3.

### Microarray analysis

Total RNA was extracted from 60 to 80 mg of frozen hypothalamic tissue blocks using an RNeasy Mini Kit with on-column DNase treatment (Qiagen). RNA quality was measured using a Bioanalyzer 2100 (Agilent Technologies). For microarray (Agilent-028279 SurePrint G3 Rat GE 8 × 60 K Microarray, Agilent Technologies), RA injected samples (n = 4) and control samples (n = 4) were analysed with dye-swap. Labelling and hybridisation of the RNA samples was outsourced to the Genomics Laboratories at the Rowett Institute of Nutrition and Health. Briefly, 100 ng of total RNA was used with the Low Input Quick Amp Labelling kit, two-colour (Agilent Technologies). RNA was amplified in two steps to cRNA, incorporating Cy-3 and Cy-5 dyes, and was purified with RNeasy mini spin columns (Qiagen). For hybridisation, the Gene Expression Hybridisation Kit (Agilent Technologies) was used. Labelled cRNA was hybridised to SurePrint G3 Rat GE 8 × 60 K microarray slides (Agilent Technologies) rotating at 65 °C for 17 hrs. The arrays were washed using the Gene Expression Wash Buffer Kit (Agilent Technologies) and then scanned on a SureScan High Resolution Scanner (Agilent Technologies).

### *Ex-vivo* hypothalamic slice cultures and qPCR

*Ex-vivo* hypothalamic slice cultures were set up as previously described^9^. Slices were treated for 48 hrs with 1 μM RA or an equivalent concentration of DMSO (0.1%) diluted in culture medium. Following treatment, total RNA was extracted from slices using a Qiagen RNeasy RNA purification kit. cDNA was synthesized from 500 ng total RNA using a High Capacity RNA-to-cDNA Master Mix (Applied Biosystems). qPCR reactions were set up using SensiMix SYBR master mix (Bioline) and run on a Roche LightCycler 480. Primer sequences for *Rarres2* were F-CGGACATACACGGGACAGAGCTTGA and R-CAGCTGAGAAGAACAGGTCATCAGCAC and for *Actb* were F-CCACACCCGCCACCAGTTCG and R-TACAGCCCGGGGAGCATCGT. *Rarres2* expression was normalised to *Actb* levels. Standard curves and blank controls were run for both sets of primers.

### *In-situ* hybridisation

F344 rat hypothalamus coronal sections were cut and *in-situ* hybridisation was performed as described previously^40^. Riboprobe templates for *Rarres2*, *Cmklr1*, *Gpr1* and *vimentin* were amplified from rat hypothalamic cDNA and *Ccrl2* from rat muscle cDNA. Primers for *Rarres2* were F-TGGGCACAGCGGACATACACG and R-ACCATCCGGCCTAGAACTTTACCC; for *Cmklr1* were F-CGGGAAAGCCATGTGCAAGATTAG and R-AGAGGAAGCGGGTGACGGTGAC; for *Gpr1* were F-CGGGAAAGCCATGTGCAAGATTAG and R-AGAGGAAGCGGGTGACGGTGAC; for *Ccrl2* were F-TTGCCCGAAGCTGTGTTTTACGAG and R-GAGGAGCGGGTTGACACAGCAGT and for *Vimentin* were F-AAGGCCCGTGTCGAGGTGGAGAG and R-TGGCGCAGGGCATCGTTCTTC. *Rarres2*, *Grp1*, *Ccrl2* and *vimentin* were cloned into Strata Clone Blunt PCR Cloning Vector (Stratagene), *Cmklr1* was cloned into pCR4 Blunt TOPO vector (Invitrogen). Riboprobe templates were prepared as described earlier for growth hormone-releasing hormone (Ghrh) and somatostatin (Srif)^40^, POMC and AgRP^43^ and NPY^44^. ^35^S-labelled antisense riboprobes were generated from ~150 ng of amplified inserts by T3 or T7 RNA polymerase as appropriate.

### Immunohistochemistry

Brains were removed from perfused F344 rats, post-fixed overnight in 4% paraformaldehyde/0.1 M PB, cryoprotected with 30% sucrose/PB before snap frozen in isopentane on dry ice and stored at −70 °C. 30 μm thick coronal brain sections were cut on a cryostat, mounted on Superfrost^®^Plus slides (Thermo Fisher Scientific) and stored in PBS/0.02% NaN_3_ at 4 °C. Immunohistochemistry was carried out using standard protocols. Briefly, slides were washed in 1xTBS, blocked in 5% bovine serum albumin/TBS/0.5% Tween 20 for 1 hr and incubated in primary antibodies, chicken anti-vimentin (1:1000; Millipore) and rabbit anti-glial fibrillary acidic protein (GFAP; 1:1000, Millipore) at 4 °C overnight. Immunoreactivity was visualised using secondary antibodies conjugated to fluorescent dyes (anti-chicken IgY, 1:200, R&D Systems and Alexa Fluor^®^488, anti-rabbit IgG, 1:200, Molecular Probes). A nuclear stain was performed by mounting the slides with Vectashield^®^Mounting Medium with DAPI (Vector Laboratories).

### Combined *in-situ* hybridisation and immunohistochemistry

Rarres2 riboprobes were DIG labelled by incubating 100 ng PCR amplified template for 2 hrs at 37 °C, followed by DNase RQ1 (Promega) incubation for 15 min at 37 °C. The riboprobe was purified using a ProbeQuant G50 column (Amersham). Labelling efficiency was tested by membrane dot-blotting and processing with anti-DIG-POD Fab fragments and DAB substrate (Roche Diagnostics). For *in-situ* hybridisation, slides were prepared as described. Prior to primary antibody incubation, slides were treated with 0.3% H_2_O_2_ before being blocked with 2% TSA detection system blocking reagent (Molecular Probes), followed by Streptavidin/Biotin blocking reagent (Vector labs). Slides were incubated in anti-DIG-POD fab fragments in 2% TSA detection system blocking reagent (1 in 100) overnight at 4 °C in a humidity chamber, then washed 3× for 5 min in 100 mM Tris/HCl, 150 mM NaCl, 0.3% Tween-20. Slides were incubated in Tyramide 488 (Molecular Probes) for 2 hrs in the dark and then washed 3× for 5 min in 100 mM Tris/HCl, 150 mM NaCl, 0.3% Tween-20. Slides were subsequently processed for detection of Vimentin immuno-reactivity following the protocol described above.

### Statistics

Microarray data were analysed within the statistical programming environment R (version 3.02) using the Bioconductor library limma (3.18.9)^45^. Loess normalisation was used to eliminate intensity depending dye-effects and the normalised data were analysed on a log 2-scale. Identical probes represented by the same oligo were averaged within arrays. Comparisons between RA treatment and controls where conducted by the limma-specific moderated t-test which borrows information across probes to stabilize the estimation of standard errors^46^. Dye-swaps were used as a blocking variable in this analysis to account for the re-use of the same samples with changed Cy3-Cy5 setting. This analysis provides a *p*-value and an average log 2-fold change for each pairwise comparison of interest. For pathway analysis, the Gene Ontology (GO) database was used to find groups of genes with similar functions. The number of genes whose annotation corresponded to a GO term was calculated as well as the number of differentially expressed genes among them if their *p*-value was below 0.01. Fisher’s exact test was used to test whether the number of differential genes within a GO term exceeded what could be expected by chance, which yields a *p*-value on GO term/pathway level. Data sets are accessible at the Gene Expression Omnibus website [GSE65300].

All other data were analysed by a two-tailed Student’s t-test, one-way ANOVA or two-way RM ANOVA (treatment group x time interaction) followed by a Tukey’s post-hoc test using Sigma Plot statistical software. The results are presented as means ± SEM and differences were considered significant if *p* < 0.05.

## Additional Information

**How to cite this article**: Helfer, G. *et al.* A neuroendocrine role for chemerin in hypothalamic remodelling and photoperiodic control of energy balance. *Sci. Rep.* **6**, 26830; doi: 10.1038/srep26830 (2016).

## Figures and Tables

**Table 1:** Pathway analysis of genes found by microarray showing the strongest difference after injection of RA.

Pathway	Annot.genes	Sig. genes	*p*value*
inflammatory response	152	50	1.36 × 10^−17^
immune response	197	50	7.14 × 10^−14^
positive regulation of I-κB kinase/NF-κB cascade	130	33	1.03 × 10^−9^
antigen processing and presentation	54	21	2.41 × 10^−9^
antigen processing and presentation of exogenous peptide antigen via MHC class II	17	13	6.76 × 10^−9^
chemotaxis	53	20	8.65 × 10^−9^
innate immune response	62	20	7.33 × 10^−8^
ruffle	58	19	1.26 × 10^−7^
immunoglobulin mediated immune response	15	11	1.34 × 10^−7^
Ras protein signal transduction	60	19	1.95 × 10^−7^
chemokine activity	39	15	4.97 × 10^−7^
neutrophil chemotaxis	28	13	5.27 × 10^−7^

The most significant changes were in genes related to inflammatory and immune responses. The 12 pathways with the highest percentage of changes are shown. *p value calculated by Fisher test.
